# Fish Probiotics: Cell Surface Properties of Fish Intestinal Lactobacilli and *Escherichia coli*

**DOI:** 10.3390/microorganisms11030595

**Published:** 2023-02-27

**Authors:** Susanna Mirzabekyan, Natalya Harutyunyan, Anahit Manvelyan, Lilit Malkhasyan, Marine Balayan, Shakhlo Miralimova, Michael L. Chikindas, Vladimir Chistyakov, Astghik Pepoyan

**Affiliations:** 1 Division of Food Safety and Biotechnology, Armenian National Agrarian University, Yerevan 0009, Armenia; 2 Institute of Microbiology, Academy of Sciences of the Republic of Uzbekistan, Tashkent 100125, Uzbekistan; 3 Health Promoting Natural Laboratory, Rutgers State University, New Brunswick, NJ 08901, USA; 4 Center for Agrobiotechnology, Don State Technical University, 344002 Rostov-on-Don, Russia; 5 Department of General Hygiene, I.M. Sechenov First Moscow State Medical University, Bolshaya Pirogovskaya Str., 19/1, 119146 Moscow, Russia; 6 D.I. Ivanovsky Academy of Biology and Biotechnology, Southern Federal University, Prosp. Stachky 194/1, 344090 Rostov-on-Don, Russia; 7 The International Scientific-Educational Center of the National Academy of Sciences of the Republic of Armenia, Yerevan 0019, Armenia

**Keywords:** fish, probiotic, *E. coli*, lactic acid bacteria, cell surface hydrophobicity, auto-aggregation, biofilm formation ability

## Abstract

The properties of intestinal bacteria/probiotics, such as cell surface hydrophobicity (CSH), auto-aggregation, and biofilm formation ability, play an important role in shaping the relationship between the bacteria and the host. The current study aimed to investigate the cell surface properties of fish intestinal bacteria and probiotics. Microbial adhesion to hydrocarbons was tested according to Kos and coauthors. The aggregation abilities of the investigated strains were studied as described by Collado and coauthors. The ability of bacterial isolates to form a biofilm was determined by performing a qualitative analysis using crystal violet staining based on the attachment of bacteria to polystyrene. These studies prove that bacterial cell surface hydrophobicity (CSH) is associated with the growth medium, and the effect of the growth medium on CSH is species-specific and likely also strain-specific. Isolates of intestinal lactobacilli from fish (*Salmo ischchan*) differed from isolates of non-fish/shrimp origin in the relationship between auto-aggregation and biofilm formation. Average CSH levels for fish lactobacilli and *E. coli* might were lower compared to those of non-fish origin, which may affect the efficiency of non-fish probiotics use in fisheries due to the peculiarities of the hosts’ aquatic lifestyles.

## 1. Introduction

Fish are an important component of aquaculture. The productivity of fish aquaculture is most dependent on the effective control of emerging fish diseases, which, in recent decades, has led to the widespread utilization of antimicrobials. This will eventually lead to the development and spread of antimicrobial-resistant pathogens [1,2,3]. Therefore, there is an urgent need to develop alternative methods for combating fish pathogens, reducing the accumulation of antibiotic residues in fish meat, and other related environmental problems [4]. Such methods include phage [5,6] and probiotic therapies [7,8,9,10,11,12]. According to the International Scientific Association for Probiotics and Prebiotics, probiotics are live microorganisms that, when administered in adequate amounts, confer a health benefit to the host (https://isappscience.org/for-scientists/resources/probiotics) (accessed on 17 November 2019) [13]. The host might be a human [14,15,16], animal [17], plant, or soil [18]. In addition to lactobacilli, *Escherichia coli* (*E. coli*) are widely used as probiotics [19,20,21,22]. Although pathogenic strains of *E. coli* cause infections, commensal *E. coli* are an important component of the human and animal gut microflora [23,24,25,26,27,28,29].

The mechanisms of action of probiotics in aquaculture include the secretion of antagonistic compounds [30,31], effects on quorum sensing mechanisms [32,33], inhibition of adhesion and colonization by pathogenic bacteria via competitive exclusion [34], modulation of gut microbiota and immune reactions [35], antiviral effects [36], and the improvement of water quality and modulation of the aquatic microbiota [37,38]. Considering the variety of different mechanisms of action for probiotics, the most important question is whether candidate probiotic strains exhibit one or more of these specific beneficial properties and how to identify and select the most useful strains for use in freshwater fish aquaculture [39,40,41].

Research on fish microbiomes is essential for the accumulation of important data required to develop targeted probiotics. It is known that the microbiome of the skin is vital to fish growth, behavior, digestion, immune health, and overall physiology, gills, and gut [42]; it is also dependent on host genetics, developmental stage, diet, and habitat [43]. A retrospective analysis of changes in the bacterial flora of fish and water for the period 1979–2014 showed that after the transition from pond technologies to industrial methods, anaerobic aeromonads and non-fermenting alkaline formers became the predominant organisms in the microbiocenosis of salmon and sturgeon fish. The study of the water microbiocenosis showed an increase in the total microbial number and the percentage of bacteria belonging to the *E. coli* group, aeromonads, acinetobacteria, moraxella, proteus, and myxobacteria [44]. Highly diverse microorganisms, which belong to Proteobacteria, Firmicutes, and Bacteroidetes, represent about 90% of the fish gut microbiota [45].

It is known that the probiotic potential of bacterial strains is closely related to cell surface characteristics such as auto-aggregation capacity, cell surface hydrophobicity, and biofilm formation ability. These cell surface properties are widely used to characterize and screen probiotic strains in vitro [46,47]. The ability to adhere to the intestinal epithelium is one of the main criteria when choosing probiotics. This ability may increase the chances of probiotics surviving in the gastrointestinal tract and thus allow the bacteria to exert positive health effects [48]. The first stage of adhesion seems to be auto-aggregation, which results in the formation of a barrier and prevents the adhesion of other bacteria [49]. An important property affecting the ability of bacteria to adhere is the hydrophobicity of the cell surface. It has been shown that bacteria with higher hydrophobicity can better adhere to epithelial cells [50]. Falah and coauthors believe that hydrophobicity is one of the important properties that improve the first contact between bacteria and host cells and that the study of hydrophobicity can be used as a preliminary test of the ability of probiotic bacteria to adhere to epithelial cells [51]. Thus, auto-aggregation, coaggregation, and surface hydrophobicity are considered to be important characteristics that provide potential benefits for microorganisms during colonization of the intestinal tract [52]. At the same time, biofilms are a form of cell immobilization resulting from the attachment of microbes to solid carriers. Biofilms allow the bacteria in the biofilm to withstand various stresses such as pH changes or starvation [53,54].

Given the above, and the fact that an aquatic lifestyle is able to influence the required indexes of probiotics made for aquatic and terrestrial animals, the aim of this study was to investigate the cell surface properties of bacterial isolates from fish and probiotics of human, animal, and fish origins that may stimulate the growth of fish and help control pathogens. The main objective is to clarify if there are any differences between the cell surface properties of gut bacteria of fish and non-fish origins, particularly regarding cell surface hydrophobicity, biofilm formation, and auto-aggregation abilities, which can play an important role in the productive utilization of various probiotics in fish farming. 

## 2. Materials and Methods

### 2.1. Isolation of Lactic Acid Bacteria and E. coli Strains

The predominant cultivable isolates from the 51 randomly chosen male *Salmo ischchan aestivalis* and 47 male *Salmo ischchan gegarkuni* (weight: 150–200 g) from various local fish farms in different regions of Armenia (Gegharkunik, Ararat, and Armavir regions) were investigated. The samples were received from humanely euthanized fish. The isolates from the most diluted samples were *Escherichia coli* and lactobacilli. One *Enterococcus* strain was found among the predominant cultivable fish isolates. Fish from the farms were transported in thermal bags to the ANAU laboratory and processed within 2 h of acquisition. The entire intestines were isolated according to Floris and coauthors [55]. Samples were added to 0.1% (w/v) peptone and incubated at 37 °C overnight to enrich the number of microbes. Overnight samples were serially diluted 10-fold, then spread on deMan Rogosa and Sharpe agar (MRS) (Thermo Scientific Oxoid, Waltham, MA, USA) for the detection and enumeration of *Lactobacillus* and on Endo agar (Thermo Scientific Oxoid, Waltham, MA, USA) for the isolation and differentiation of *E. coli*. Plates were incubated at 37 °C for 24 h.

A total of 15 commensal *E. coli* and 5 *Lactobacillus* spp. isolates, all randomly selected and morphologically different, and *Enterococcus* spp. were investigated.

### 2.2. Identification of Fish Lactobacilli and E. coli Isolates

The bacterial isolates were characterized based on gram staining, morphology observation according to Bergey’s manual of determinative bacteriology, and further confirmation by PCR. The isolates were cultured in Luria Bertani Broth, Miller (HiMedia, Maharashtra, India), at 37 °C for 24 h, and bacterial genomic DNA was extracted using QIAamp DNA Micro Kit (Qiagen, Hilden, Germany).

For the identification of *E. coli*, one microliter of the DNA-containing elution buffer was used for the PCR. The oligonucleotide primers (Integrated DNA Technologies, Inc., Coralville, IA, USA) used to detect *E. coli* were coliF (5′-CCG ATA CGC TGC CAA TCA GT-3′) and coliR (5′-ACG CAG ACC GTA GGC CAG AT-3′). PCR was performed using a Thermal Cycler BioCycler TC-S (Boeco, Hambur, Germany), GoTaq^®^ Green Master Mix, 2X (Promega Corporation, Fitchburg, MA, USA), and using the following program: 5 min initial denaturation at 95 °C; 30 cycles of denaturation (30 s at 94 °C), annealing (30 s at 56 °C), and extension (30 s at 72 °C); a final extension at 72 °C for 5 min. The amplification products were visualized under a UV trans-illuminator (Vilber Lourmat ECX-15.M, Collégien, France). Ready-to-use PCR Kits (K792 *Escherichia coli* double-check, Genekam Biotechnology AG, Collégien, Germany) were used for the identification of *E. coli* isolates.

*Enterococcus* spp. were identified using a BactoReal^®^ Kit *Enterococcus* spp. (Ingenetix GmbH, Vienna, Austria).

*Lactobacillus* strains were confirmed using Forw R16-1 (5′-CTT GTA CAC ACC GCC CGT CA-3′) and Rev LbLMA1 (5′-CTC AAA ACT AAA CAA AGT TTC-3′) primers (Integrated DNA Technologies, Inc., Coralville, IA, USA). GoTaq^®^ Green Master Mix, 2X (Promega Corporation, Fitchburg, MA, USA) was used. The amplification program was 95 °C for 10 min; 35 cycles of 95 °C for 1 min, 50 °C for 1 min, and 72 °C for 1 min; and finally 72 °C for 10 min.

### 2.3. Probiotic Strains 

Probiotic bacterial strains of fish/shrimp origin from the microbial collections of the Southern Federal University of Russia (*Bacillus subtilis* str. 1R, *Bacillus amyloliquefaciens* str. 4R, *Bacillus amyloliquefaciens* str. 5R and *Bacillus cereus* str. 6R) and the Institute of Microbiology of the Academy of Sciences of the Republic of Uzbekistan (*Lactococcus* str. UZ-1, *Lactiplantibacillus plantarum* str. R3, *Lactococcus* str. UZ-2, *Enterococcus faecium* str. R2 and *Pediococcus acidilactici* str. N) were used in this study. The cell surface properties of biofilm formation ability, cell surface hydrophobicity, and auto-aggregation abilities were not assessed for the above-mentioned probiotics before the current investigations.

Probiotic strains of human/sheep/milk origin from the bank of the International Association for Human and Animals Health of Armenia (*Lacticaseibacillus rhamnosus* str. Vahe, *Lactiplantibacillus plantarum* str. ZPZ, *Lacticaseibacillus rhamnosus* str. E5-2, *Lactiplantibacillus plantarum* str. K1-3, *E. coli* str. ASAP-1 and *E. coli* str. ASAP 2-1) and Vitamax LLC, Armenia (*L. acidophilus* Er-2 str. 317/402 from the commercial probiotic “Narum Caps”, *L. acidophilus* Er-2 str. 317/402 from the commercial synbiotic “NARUM CAPS FAST” and commercial synbiotic “NARUM TAB”, https://mynarum.com/ (accessed on 28 December 2021)) were also used.

### 2.4. Cell Surface Properties

#### 2.4.1. Bacterial Cultures

The lactic acid bacteria (LAB) strains were grown in MRS broth at 37 °C for 48 h. The *E. coli* strains were grown in Luria Bertani (LB) broth at 37 °C for 24 h. *Bacillus* spp. were grown in MRS broth at 37 °C for 48 h.

For studying the effect of growth medium on bacterial cell surface properties, bacterial cultures were also grown in a mixed medium containing MRS and LB in a ratio of 1:3. Based on several trials, a 1:3 (MRS and LB) mixed medium was chosen as it supported the growth of all tested bacteria.

Cultures were centrifuged (1165× *g* for 15 min), washed twice, and resuspended in sterile phosphate-buffered saline (PBS, pH 7) to an optical density matching a 0.5 MacFarland standard (OD_600_) to standardize the bacterial density at 10^8^ CFU/mL. The OD_600_ of each bacterial suspension (BS) was measured using a spectrophotometer (Stat Fax 3300, Awareness Technology, Palm City, FL, USA).

#### 2.4.2. Cell Surface Hydrophobicity

Microbial adhesion to hydrocarbons was tested according to Kos and coauthors [56] with a slight modification: xylene was used as the hydrocarbon solvent in this test. Bacterial cultures were adjusted to optical density OD_600_ = 0.5 (the number of *Lactobacillus* sp. and *Escherichia* sp. is 10^11^ bacteria/mL of culture medium, and the number of *Bacillus* sp. is 10^8^ bacteria/mL). Then, 1 mL of xylene was added to 1 mL of the bacterial suspension. After a 10 min incubation at room temperature, the two-phase suspension was mixed by vortexing for 2 min and incubated for an additional 20 min at room temperature. The hydrocarbon layer was removed completely, and the absorbance of the aqueous-phase cell suspension was measured at 600 nm (Stat Fax 3300, Awareness Technology). The cell surface hydrophobicity (CSH) was expressed as a percentage using the following formula:

$$\mathrm{CSH}=\left(1-\frac{\mathrm{ODA}}{\mathrm{ODB}}\right)*100\;\%$$

where ODB and ODA are the absorbances of the bacterial suspension before and after mixing with hydrocarbon, respectively.

#### 2.4.3. Auto Aggregation Study

The aggregation abilities of the investigated strains were determined as described by Collado and coauthors [57]. The optical density (OD_600_) of the homogenized bacterial suspension was recorded and then monitored again, 2 and 24 h after incubation at 37 °C under static conditions. The percent of aggregation was evaluated as follows:

$$A=\left(1-\frac{A_{time}}{A_{0}}\right)*100\;\%$$

where *A_time_* represents the absorbance of the mixture at 2 and 24 h, and *A*_0_ is the absorbance at the starting point.

#### 2.4.4. Biofilm Formation

The ability of bacterial isolates to form a biofilm was determined by performing a qualitative analysis using crystal violet staining based on the attachment of bacteria to the surface of polystyrene [58]. Specifically, 200 µL of bacterial suspensions (OD_600_ = 0.5), incubated overnight, was transferred into a polystyrene 96-well plate (Biomat, Ala, Italy) and incubated for 48 h at 37 °C. Then, 25 µL of 0.5% crystal violet was added to each well, and the plates were incubated for 15 min at room temperature. Next, the wells’ contents were aspirated, and the empty wells were washed 3 times with PBS. Crystal violet extraction was performed using 96% ethanol, and biofilm formation abilities were evaluated photometrically at 540 nm (Stat Fax 2100, Awareness Technology, Perchtoldsdorf, Austria).

### 2.5. Statistical Analysis

All data obtained from the five independent experiments are expressed as mean ± standard deviation (SD). A *t*-test was performed at a 95% confidence interval in order to determine the statistical significance (*p* < 0.05). The results were confirmed by the Mann–Whitney test. The impact of growth medium on bacterial cell surface hydrophobicity was also evaluated by the chi-squared test, and Pearson’s correlation statistics were applied to describe correlations between the bacterial membrane characteristics (excel 2016).

## 3. Results

### 3.1. Cell Surface Hydrophobicity 

#### 3.1.1. Bacteria Grown in MRS/LB Mixed Growth Medium

There were no significant differences in the levels of cell surface hydrophobicity between the fish probiotic strains of Bacillus and LAB origins (*p* > 0.05). According to Table 1, the levels of cell surface hydrophobicity of the investigated probiotic LAB and *Bacillus* spp. from the fish and shrimp gut microbiota and of the fish LAB isolates were statistically lower than that of the lactic acid probiotics of human/sheep/milk origin (0.14 ± 0.4% vs. 8.5 ± 6.7%, *p* < 0.05) (Table 1). There were also no differences between the cell surface hydrophobicity levels of fish LAB and Bacillus isolates/probiotics and fish LAB and *Bacillus* isolates (0.14 ± 0.4% vs. 0.15 ± 0.56%, *p* > 0.05) (Table 1).

As expected, the levels of cell surface hydrophobicity of the fish *E. coli* isolates were lower than those of the probiotics of human origin (0.01 ± 0.05% vs. 4.5 ± 2.4%, *p* < 0.05 (Table 1) (usually to screen probiotics, the hydrophobicity/biofilm formation ability is taken into account [46]).

#### 3.1.2. Bacteria Grown in LB and MRS Growth Media

According to Table 1, the levels of cell surface hydrophobicity of the investigated probiotic LAB and *Bacillus* spp. isolated from the fish/shrimp gut microbiota and of the fish LAB isolates were statistically lower than those of the lactic acid probiotics of human/sheep/milk origin (1.11 ± 2.8% vs. 6.7 ± 8.25%, *p* < 0.05) (Table 1). There were no differences between the cell surface hydrophobicity levels of fish LAB and *Bacillus* isolates/probiotics and fish LAB and *Bacillus* isolates (1.11 ± 2.8% vs. 2.39 ± 3.9%, *p* > 0.05) (Table 1).

As expected, the levels of cell surface hydrophobicity of the fish *E. coli* isolates were lower than those of the *E. coli* probiotics from the gut microbiota of non-fish origin (1.07 ± 2.4% vs. 13.9 ± 4.8%, *p* < 0.05) (Table 1). Additionally, the levels of cell surface hydrophobicity of the fish *E. coli* isolates were lower than those of sheep isolates with the lowest cell surface hydrophobicity of the non-fish isolates (1.07 ± 2.4% vs. 5.17 ± 1.15%, *p* < 0.05) (Table 1). Overall, the average levels of cell surface hydrophobicity for the fish lactobacilli and *E. coli* were lower than those of non-fish origin (Table 1).

#### 3.1.3. Comparison of the Bacterial Cell Surface Hydrophobicity in Different Growth Media

Significant differences in hydrophobicity percentages were shown both for probiotics *Laticaseibacillus rhamnosus *str. Vahe, *Lactiplantibacillus plantarum* str. K1-3, *E. coli* str. ASAP-1 and *E. coli* str. ASAP-2-1 (Table 2), as well as for the strains *Lactobacillus* str. 18-3, *Enterococcus* str. 9-3, *E. coli* str. 5-1, *E. coli* str. 5-4, *E. coli* str. 5-5, and *E. coli* str. 9-2 when growing bacteria in different media.

As expected, the effect of growth medium on bacterial surface hydrophobicity characteristics was shown to be species-specific and probably also strain-specific. As can be seen in the table, the hydrophobicity of the probiotic strain *Lacticaseibacillus rhamnosus *str. Vahe in the mixed MRS/LB medium was lower than that in the MRS medium (4.5 ± 3.5% vs. 19.6 ± 7.8%, *p <* 0.05), whereas *Lactiplantibacillus plantarum* str. K1-3 cells were more hydrophobic when grown in the mixed MRS/LB medium (9.2 ± 7.1% vs. 2.2 ± 1.6%, *p <* 0.05). *L. acidophilus* str. Er-2 strain 317/402 from the probiotic formulation Narum Caps exhibited the highest cell surface hydrophobicity in comparison with the same strains isolated from synbiotic formulations (19.3 ± 6.2% vs. 7 ± 5.3% and 15.6 ± 5.4%, *p <* 0.05) (Table 2). In addition, the growth medium might affect the strains to varying degrees. For example, *Lactiplantibacillus plantarum* str. ZPZ shows 2.5 ± 4.5% and 1.8 ± 2.2%, and the strain K1-3 shows 9.2 ± 7.1% and 2.2 ± 1.6% in MRS/LB medium (1:3 ratio) and MRS medium, accordingly.

There were no significant differences in the levels of cell surface hydrophobicity between the fish *E. coli* and LAB isolates and the Bacillus and LAB probiotics (*p* > 0.05) (Table 1). However, we did not take into account the cell surface properties of candidate probiotics when screening fish-, *Bacillus*-, and LAB-origin probiotics from the fish bacteria, which, perhaps, may have affected the conclusion concerning fish probiotics. LAB and *E. coli* isolated from the gut of *Salmo ischchan* had lower CSH levels than gut bacteria of non-fish/shrimp origin.

### 3.2. Biofilm Formation Ability

The results on bacterial biofilm formation abilities are given in Table 3. In contrast to the data obtained on cell surface hydrophobicity, biofilm formation abilities were significantly different for the fish probiotic strains of *Bacillus* and LAB origin (1.93 ± 0.87 D vs. 0.139 ± 0.035 D, *p* < 0.05). The average of fish Bacillus biofilm formation abilities was also higher than that of the LAB probiotics of non-fish origin (1.93 ± 0.87 D vs. 0.169 ± 0.01 D; *p* < 0.05). Moreover, according to Table 3, no significant differences were found in the fish LAB probiotics in comparison with the human/sheep/milk probiotics in terms of biofilm formation ability (0.139 ± 0.02 D vs. 0.169 ± 0.01 D, *p* > 0.05).

Simultaneously, there were no differences between the bacterial biofilm formation abilities of fish LAB probiotics and fish LAB isolates (0.139 ± 0.035 D vs. 0.228 ± 0.07 D; *p* > 0.05) (Table 3). Even though fish *E. coli* isolates were statistically different from human-origin probiotic *E. coli* strains (average biofilm formation ability of all fish *E. coli* isolates vs. average biofilm formation ability of probiotic *E. coli* strains, as 0.24 ± 0.5 D vs. 1.02 ± 0.26 D; *p* < 0.05) (Table 3), the biofilm formation ability was evaluated to be the same as that of non-fish origin isolates of *E. coli*. An exception was found for one isolate, whose biofilm formation ability was statistically higher compared to other fish *E. coli* (1.021 ± 0.25 D vs. 0.24 ± 0.5 D, *p* < 0.05). No differences were observed in the degree of biofilm formation of the studied bacteria when grown in a mixed medium.

### 3.3. Bacterial Cell Auto-Aggregation

The results of cell aggregation in LB and MRS growth media are given in Table 4. Interestingly, the cell aggregation of fish *E. coli* did not have any describable specificity in comparison with that of probiotic *E. coli* strains (54.43 ± 8.41% vs. 57.45 ± 3.97%, *p* > 0.05). In comparison, the cell aggregation for fish LAB strains was statistically lower that of probiotic strains (61.02 ± 8.32% vs. 94.08 ± 3.33% and 95.67 ± 2.6%, *p* > 0.05). The *Enterococcus 9-3* strain has the same level of auto-aggregation as the probiotic strain *Enterococcus faecium* R2.

The highest Pearson correlations were shown between biofilm formation ability and surface hydrophobicity, biofilm formation and auto-aggregation abilities, and auto-aggregation and biofilm formation abilities for the *E. coli* probiotics with non-fish origin (|r| = 1). These associations were comparably weak in fish *E. coli* isolates. Interestingly, non-fish origin lactobacilli isolates and probiotics had a weak correlation between their cell surface hydrophobicity and auto-aggregation, and cell surface hydrophobicity and biofilm formation. However, the correlation between the auto-aggregation and biofilm formation was high for the fish lactobacilli (|r| = 0.982), which, against the background of the same ability to form biofilms, was probably due to the relatively low auto-aggregation of these lactobacilli (Table 5).

A weak relation was also discovered between the auto-aggregation and biofilm formation abilities of fish *Bacillus* spp. Other associations related to fish bacteria were even weaker (|r| < 0.2).

## 4. Discussion

### 4.1. Cell Surface Hydrophobicity

It is well known that the growth medium affects the ability of lactobacilli to form biofilms [61,62]. The hydrophobicity of the cell surface determines the ability of bacteria to attach to the cell, although physical parameters such as Brownian motion, van der Waals attraction, gravitational forces, and the surface electrostatic effect cannot be ignored [63].

The present studies confirm that the hydrophobicity of the cell surface of the studied LAB, as well as *E. coli* cells, depends on the growth medium. However, no correlation between hydrophobicity and growth medium was found; the results were specific for different species (also probably for strains).

Various methods are used to regulate the hydrophobicity of the cell surface of probiotics. It can be assumed that the targeted selection of a growth medium for probiotics may be one of the major approaches for this purpose. It is possible that modulation of the cell surface hydrophobicity of probiotic bacteria by prebiotics can determine the effectiveness of synbiotic preparations.

It is also interesting that fish intestinal bacteria, particularly lactobacilli and *E. coli*, have low cell surface hydrophobicity, regardless of the growth medium (Table 1). In order to understand this phenomenon, additional studies will be required to elucidate the mechanisms of host–bacteria interaction in fish, as well as the influence of the environment on the fish intestinal microflora. The surface proteins of LAB can also affect the hydrophobic characteristics of the cell surface and are important in the processes of adaptation of the biophysical characteristics of the cell surface in response to environmental changes [64]. It is also known that surface proteins can participate in combating fish pathogens. For example, a protein extract can inhibit the adhesion of the pathogen to epithelial cells [65]. The results of our studies do not exclude the possible role of surface layer proteins in the hydrophobic characteristics of the cell surface of lactobacilli grown in various media. Based on the presented and published data, it can also be assumed that lactic acid bacteria and *E. coli* may have adaptive functions in the microflora of fish. On the other hand, the lower level of hydrophobicity of the cell surface of fish bacteria probably indicates the transitory status of these bacteria.

All the fish probiotic strains used in this study were selected as probiotics due to their antagonistic behavior towards fish pathogens [11,66]. In this case, the absence of significant differences in the levels of cell surface hydrophobicity between the fish *E. coli* and LAB isolates and Bacillus- and LAB-origin probiotics (*p* > 0.05) (Table 1) allows for the assumption that the cell surface hydrophobicity of *E. coli*, and LAB/*Bacillus* strains might not play a significant role in combating fish pathogens. On the other hand, there is a statistically significant difference between the levels of cell surface hydrophobicity of fish- and non-fish-origin probiotics (*p* < 0.05) (Table 1). The question of whether probiotics of non-fish origin with a higher cell surface hydrophobicity and the same antagonistic quality are more advantageous than those of fish origin is unanswered and needs further clarification/investigation. Moreover, if it is recommended to use non-fish-origin probiotics which have a non-beneficial cell surface hydrophobicity, considering the factors mentioned above, it might be possible to affect the strain hydrophobicity levels with other methods, including a change of environment.

### 4.2. Biofilm Formation Ability and Auto-Aggregation

Bacterial biofilms are communities of surface-attached bacteria that express distinct properties compared to their free-living counterparts, including increased antibiotic tolerance and metabolic capabilities [67]. They play an important role in the development and functioning of the host organism and protect it against pathogens [68]. For example, the investigations by Mirani and coauthors on multispecies biofilm formation from *Pseudomonas aeruginosa*, *Staphylococcus aureus*, and *E. coli* strains showed that *E. coli* dominated during the pre-biofilm stage. The authors reported that *E. coli* adapted to a biofilm lifestyle before *S. aureus* and *P. aeruginosa*. However, after adopting a biofilm lifestyle, *P. aeruginosa* gradually came to dominate the consortia and dispersed other species. This could be explained by the ability of *P. aeruginosa* to produce cis-2-decanoic acid, which can disperse or inhibit *S. aureus* and *E. coli* biofilms [69].

The presented studies show the highest correlations between biofilm formation ability and surface hydrophobicity, biofilm formation and auto-aggregation abilities, and auto-aggregation and biofilm formation abilities for the *E. coli* probiotics with a non-fish origin, similar to the pathogenic microorganisms [70,71], which is expected if we take into account the requirements for probiotics. The results of our study on the cell surface properties of *L. acidophilus* strain INMIA 9602 Er-2 317/402 Narine are consistent with the literature data on the hydrophilic properties and poor biofilm formation ability of other *Lactobacillus* strains [71].

It is known that the surface of bacterial cells consists of many identical subunits that form a porous lattice layer. Surface layer proteins are found in many species of lactobacilli. The functions of these proteins are poorly understood, but there is evidence that some surface layer proteins have protective and enzymatic functions and can also mediate the adhesion of lactobacilli to host cells or extracellular matrix proteins [72]. 

It is possible that the biofilm formation ability, the degree of surface hydrophobicity, and auto-aggregation (the first stage of adhesion) [49,73] of fish microbiome bacteria are determined by the presence of specific proteins of the surface layer, which, in turn, may underlie the adaptive properties of fish. Probably, the specificities in cell surface and auto-aggregation properties of fish lactobacilli have a noticeable impact on fish adaptive properties. Lactobacilli are known to modify their surface structure in response to environmental factors; the correlation between auto-aggregation and biofilm formation abilities might show that both of these characteristics depend on the same physical adhesive forces.

This study on the properties of the cell surface of fish intestinal bacteria is important for determining the effectiveness of the use of probiotics in fish production and requires additional research to clarify how the characteristics of bacterial surfaces contribute to probiotic effects. This is also important for assessing the role of the bacterial factor in studies of “interacting” ecosystems [74]․

## 5. Conclusions

The properties of intestinal bacteria/probiotics, such as cell surface hydrophobicity, auto-aggregation, and biofilm formation ability, play an important role in shaping the relationship between bacteria and the host. The current investigation on bacterial surface characteristics revealed a difference between probiotics of fish and non-fish origins. Interestingly, LAB and *E. coli* isolated from the intestines of fish had a low level of cell surface hydrophobicity, which was influenced by the growth medium. *Salmo ischchan* fish intestinal lactobacilli isolates also differed from non-fish origin intestinal lactobacilli/lactobacilli probiotics by their association between the auto-aggregation and biofilm formation abilities.

The bacterial auto-aggregation (Table 4) indicates that perhaps the auto-aggregation of lactic acid bacteria, in contrast to bacterial hydrophobicity, is important in the fight against pathogens. This could also apply to *E. coli* probiotics; unfortunately, we do not have fish/aquatic *E. coli* probiotics to make a general guess. Further research will be aimed at testing this hypothesis, as well as elucidating its mechanisms. It is also interesting that non-fish origin lactobacilli isolates and probiotics also had weak associations related to auto-aggregation–cell surface hydrophobicity and cell surface hydrophobicity–biofilm formation, while the auto-aggregation–biofilm formation associations were high for the fish lactobacilli (|r| = 0.982) (Table 5). Against the background of the same ability to form biofilms (Table 3), this was probably due to the relatively low auto-aggregation of these lactobacilli (Table 4).

Unlike in other animal taxa, where host genetic factors play a central role in shaping the microbiota, the intestinal microbiota of fish is mainly determined by the environmental factors of the habitat. This, along with the results of current investigations, is important for the selection of fish probiotics and the regulation of appropriate biotechnological processes.

These investigations serve as a foundation for further, more profound studies of fish bacteria/probiotics. 

## Figures and Tables

**Table 1:** Comparative characteristics of cell surface hydrophobicity of lactic acid bacteria and *E. coli* isolated from the fish gut microbiota.

Bacteria	Isolate ^/Probiotic ^^	Source	Cell Surface Hydrophobicity ^1^, %, ± Standard Deviation	Cell Surface Hydrophobicity ^2^, %, ± Standard Deviation
LAB probiotics of non-fish origin	Probiotic	Human/sheep/milk	8.5 ± 6.7	6.7 ± 8.25
LAB and *Bacillus* isolates	Isolates and probiotics	Fish/shrimp origin	0.14 ± 0.4 *p*_1_ < 0.05	1.11 ± 2.8 *p*_1_ < 0.05
LAB and *Bacillus* isolates	Isolates	Fish/shrimp origin	0.15 ± 0.56 *p*_1_ < 0.05 *p*_2_ > 0.05	2.39 ± 3.9 *p*_1_ < 0.05 *p*_2_ > 0.05
Lactobacilli and *E. coli* isolates	Isolates	Fish/shrimp origin	0.11 ± 0.35 *p*_1_ < 0.05 *p*_2_ > 0.05	1.51 ± 2.9 *p*_1_ < 0.05 *p*_2_ > 0.05
*E. coli* probiotics	Probiotic	Human gut	4.5 ± 2.4	13.9 ± 4.8
Fish *E. coli* isolates	Isolates	Fish origin	0.01 ± 0.05 *p*_3_ < 0.05	1.07 ± 2.4 *p*_3_ < 0.05
* *E. coli* isolates	Isolates	Non-fish origin	It has not been investigated	5.17 ± 1.15 *p*_4_ < 0.05
* *E. coli* and lactobacilli	Isolates	Non-fish origin	It has not been investigated	3.73 ± 1.17 *p*_4_ < 0.05

^1^ Bacterial growth medium—MRS/LB medium (1:3 ratio). ^2^ Bacteria were grown in the growth medium: *E. coli* (LB medium); LAB and *Bacillus* (MRS broth). ^ Fifteen commensal *E. coli* and four lactobacilli isolates from the fish gut microbiota, all randomly selected and morphologically different, were used in this study. ^^ Probiotic bacterial strains of fish/shrimp origin from the microbial collections of the Southern Federal University of Russia (*Bacillus subtilis* str. 1R, *Bacillus amyloliquefaciens* str. 4R, *Bacillus amyloliquefaciens* str. 5R and *Bacillus cereus* str. 6R) and the Institute of Microbiology of the Academy of Sciences of the Republic of Uzbekistan (*Lactococcus* str. UZ-1, *Lactiplantibacillus plantarum* str. R3, *Lactococcus* str. UZ-2, *Enterococcus faecium* str. R2 and *Pediococcus acidilactici* str. N) were used in this study. Probiotic strains of human/sheep/milk origin from the bank of the International Association for Human and Animals Health of Armenia (*Lacticaseibacillus rhamnosus* str. Vahe, *Lactiplantibacillus plantarum* str. ZPZ, *Lacticaseibacillus rhamnosus* str. E5-2, *Lactiplantibacillus plantarum* str. K1-3, *E. coli* str. ASAP-1 and *E. coli* str. ASAP 2-1) and Vitamax LLC, Armenia (*L. acidophilus* Er-2 str. 317/402 from the commercial probiotic “Narum Caps”, *L. acidophilus* Er-2 str. 317/402 from the commercial synbiotic “NARUM CAPS FAST” and commercial synbiotic “NARUM TAB”, https://mynarum.com/ (accessed on 28 December 2021)) were also used. * Predominated gut isolates from the sheep with the lowest cell surface hydrophobicity [59]. *p*_1_—comparison with the human/sheep/milk LAB probiotics (and *Bacillus* strains). *p*_2_—comparison of isolates and all of fish/shrimp LAB and *Bacillus* isolates. *p*_3_—comparison of fish isolates and probiotics of human origin (*E. coli*). *p*_4_—comparison of fish isolates and isolates of non-fish origin.

**Table 2:** Comparative characteristics of bacterial cell surface hydrophobicity levels in different growth media.

Bacteria	Isolate/Probiotic	Source	Cell Surface Hydrophobicity ^1^, %, ± Standard Deviation	Cell Surface Hydrophobicity ^2^, %, ± Standard Deviation
*Lacticaseibacillus rhamnosus* str. Vahe	Probiotic	Human gut	4.5 ± 3.5	19.6 ± 7.8 *p* < 0.05
*Lacticaseibacillus rhamnosus* str. E5-2	Probiotic	Human gut	1.6 ± 1.4	3.7 ± 3.3 *p* < 0.05
*Lactiplantibacillus plantarum* str. ZPZ	Probiotic	Human gut	2.5 ± 4.5	1.8 ± 2.2 *p* < 0.05
*Lactiplantibacillus plantarum* str. K1-3	Probiotic	Sheep milk	9.2 ± 7.1	2.2 ± 1.6 *p* < 0.05
*L. acidophilus* Er-2 str. 317/402	Synbiotic	Commercial synbiotic NARUM CAPS FAST	7 ± 5.3	It has not been investigated
*L. acidophilus* Er-2 str. 317/402	Synbiotic	Commercial synbiotic NARUM TAB	15.6 ± 5.4	It has not been investigated
*L. acidophilus* Er-2 str. 317/402	Probiotic	Commercial probiotic NARUM CAPS	19.3 ± 6.2	It has not been investigated
*E. coli* str. ASAP-1	Probiotic	Human gut	3.2 ± 2.4	10.6 ± 3.8 *p* < 0.05
*E. coli* str. ASAP-2-1	Probiotic	Human gut	5.8 ± 3.3	17.6 ± 5.2 *p* < 0.05

^1^ Bacterial growth medium—MRS/LB medium, (1:3 ratio). ^2^ Bacteria were grown in the growth medium: *E. coli* (LB medium); LAB and *Bacillus* (MRS broth). *p*—hydrophobicity levels’ comparison for different growth media.

**Table 3:** Comparative characteristics of biofilm formation ability **^X^** of lactic acid bacteria and *E. coli* isolated from the fish gut microbiota, D_average_ ± standard deviation.

Bacteria	Source	Biofilm Formation Ability
Fish *Bacillus* probiotics ^	Fish/shrimp origin	1.93 ± 0.87
Fish LAB probiotics ^2^^	Fish/shrimp origin	0.139 ± 0.035 *p*_1_ < 0.05
LAB probiotics of non-fish origin ^3^^	Human/sheep/milk	0.169 ± 0.01 *p*_1_ < 0.05 *p*_2_ > 0.05
Fish LAB isolates ^4^^	Fish/shrimp origin	0.228 ± 0.07 *p*_3_ > 0.05
*E. coli* probiotics ^3^^	Human gut	1.02 ± 0.26
Fish *E. coli* isolates ^4^^	Fish origin	0.24 ± 0.5 *p*_4_ < 0.05
* *E. coli* isolates	Non-fish origin	0.20 ± 0.91 *p*_5_ > 0.05

^X^ Bacteria were grown in the growth medium: *E. coli* (LB medium); LAB and Bacillus (MRS broth). * Predominated gut isolates from the sheep with the lowest cell surface hydrophobicity [59,60]. ^ Probiotic bacterial strains of fish/shrimp origin from the microbial collections of the Southern Federal University of Russia (*Bacillus subtilis* str. 1R, *Bacillus amyloliquefaciens* str. 4R, *Bacillus amyloliquefaciens* str. 5R and *Bacillus cereus* str. 6R) were used, ^2^^ Probiotic bacterial strains of fish/shrimp origin from the microbial collections of the Institute of Microbiology of the Academy of Sciences of the Republic of Uzbekistan (*Lactococcus* str. UZ-1, *Lactiplantibacillus plantarum* str. R3, *Lactococcus* str. UZ-2, *Enterococcus faecium* str. R2 and *Pediococcus acidilactici* str. N) were used. ^3^^ Probiotic strains of human/sheep/milk origin from the bank of the International Association for Human and Animals Health of Armenia (*Lacticaseibacillus rhamnosus* str. Vahe, *Lactiplantibacillus plantarum* str. ZPZ, *Lacticaseibacillus rhamnosus* str. E5-2, *Lactiplantibacillus plantarum* str. K1-3, *E. coli* str. ASAP-1 and *E. coli* str. ASAP 2-1) were also used. ^4^^ Fifteen commensal *E. coli* and five lactobacilli isolates from the fish gut microbiota, all randomly selected and morphologically different, were used in this study. *p*_1_—comparison with the fish *Bacillus* probiotics. *p*_2_—comparison of fish/shrimp LAB and non-fish/shrimp origin LAB probiotics. *p*_3_—comparison of fish/shrimp LAB isolates and fish/shrimp LAB probiotics. *p*_4_—comparison of fish isolates and probiotic isolates of non-fish origin. *p*_5_—comparison of fish *E. coli* isolates and isolates of non-fish origin.

**Table 4:** Auto-aggregation of lactic acid bacteria and *E. coli* isolated from the fish gut microbiota, average ± standard deviation.

Bacteria *	Source	Cell Auto-Aggregation, %
Fish LAB probiotics ^	Fish/shrimp origin	94.08 ± 3.33
LAB probiotics of non-fish origin ^2^^	Human/sheep/milk	95.67 ± 2.6 *p* > 0.05
Fish LAB isolates ^3^^	Fish/shrimp origin	61.02 ± 8.32 *p* < 0.05
*E. coli* probiotics ^2^^	Human gut	57.45 ± 3.97
Fish *E. coli* isolates ^3^^	Fish origin	54.43 ± 8.41 *p* > 0.05

* Bacteria were grown in the growth medium: *E. coli* (LB medium); LAB and Bacillus (MRS broth). ^ Probiotic bacterial strains of fish/shrimp origin from the microbial collections of the Institute of Microbiology of the Academy of Sciences of the Republic of Uzbekistan (*Lactococcus* str. UZ-1, *Lactiplantibacillus plantarum* str. R3, *Lactococcus* str. UZ-2, *Enterococcus faecium* str. R2 and *Pediococcus acidilactici* str. N) were used. ^2^^ Probiotic strains of human/sheep/milk origin from the bank of the International Association for Human and Animals Health of Armenia (*Lacticaseibacillus rhamnosus* str. Vahe, *Lactiplantibacillus plantarum* str. ZPZ, *Lacticaseibacillus rhamnosus* str. E5-2, *Lactiplantibacillus plantarum* str. K1-3, *E. coli* str. ASAP-1 and *E. coli* str. ASAP 2-1) were also used. ^3^^ Fifteen commensal *E. coli* and five *lactobacilli* isolates from the fish gut microbiota, all randomly selected and morphologically different, were used in this study. *p*—comparison with the probiotic strains.

**Table 5:** Correlations: cell surface hydrophobicity and auto-aggregation ability, biofilm formation and auto-aggregation ability.

Bacteria ^X^	Isolate/Probiotic	Pearson Correlation Coefficient, |r|	
		**CSH-AA**	**BF-AA**
Fish probiotics ^ (*Bacillus* spp.)	Probiotic	0.11	0.457
Fish lactobacilli isolates ^2^^	Isolate	0.397	0.982
Non-fish lactobacilli probiotics ^3^^	Probiotic	0.069	0.020
*E. coli* probiotics with non-fish origin ^3^^	Probiotic	1	1
Fish *E. coli* isolates ^2^^	Isolate	0.424	0.251

^ Probiotic bacterial strains of fish/shrimp origin from the microbial collections of the Southern Federal University of Russia (*Bacillus subtilis* str. 1R, *Bacillus amyloliquefaciens* str. 4R, *Bacillus amyloliquefaciens* str. 5R and *Bacillus cereus* str. 6R) were used. ^2^^ Fifteen commensal *E. coli* and five *lactobacilli* isolates from the fish gut microbiota, all randomly selected and morphologically different, were used in this study. ^3^^ Probiotic strains of human/sheep/milk origin from the bank of the International Association for Human and Animals Health of Armenia (*Lacticaseibacillus rhamnosus* str. Vahe, *Lactiplantibacillus plantarum* str. ZPZ, *Lacticaseibacillus rhamnosus* str. E5-2, *Lactiplantibacillus plantarum* str. K1-3, *E. coli* str. ASAP-1 and *E. coli* str. ASAP 2-1) were also used. ^X^ Bacteria were grown in the growth medium: *E. coli* (LB medium); LAB and Bacillus (MRS broth). CSH—cell surface hydrophobicity. BF—biofilm formation ability. AGG—auto-aggregation ability. 0.45 < |r|< 0.75—moderately correlated relationship. |r| > 0.7—a fairly strong relationship. |r| < 0.45 weak relationship. r = 0—no relationship.

## Data Availability

The original contributions presented in the study are included in the article. Further inquiries can be directed to the corresponding author.
